# Pre-event quality of life and its influence on the post-event quality of life among patients with ST elevation and non-ST elevation myocardial infarctions of a premier province of Sri Lanka

**DOI:** 10.1186/s12955-017-0730-9

**Published:** 2017-08-01

**Authors:** P. K. B. Mahesh, M. W. Gunathunga, Saroj Jayasinghe, S. M. Arnold, R. Haniffa, A. P. De Silva

**Affiliations:** 1 Office of Regional Director of Health Services, Colombo, Sri Lanka; 2 Department of Community Medicine, Faculty of Medicine, University of Colombo, Colombo, Sri Lanka; 3 Department of Clinical Medicine, Faculty of Medicine, University of Colombo, Colombo, Sri Lanka; 4 Office of Regional Director of Health Services, Colombo, Sri Lanka; 5 Mahidol Oxford Tropical Medicine Research Unit, Bangkok, Thailand; 6 Ministry of Health, Colombo, Sri Lanka

**Keywords:** Quality of Life, pre-MI QOL, post-MI QOL, STEMI, NSTEMI, Sri Lanka, SF-36

## Abstract

**Background:**

Pre-event Quality of Life (QOL) reflects the true social circumstances in which people live prior to the onset of myocardial infarctions. It is believed to be a predictor of the post-event QOL. The aim of this study was to describe the pre-event QOL and its influence on the post-event Quality of Life among patients with ST elevation (STEMI) and Non-ST elevation myocardial infarctions (NSTEMI) using Short Form-36 (SF-36), a generic QOL tool with 8 domains. Documented literature is rare in this regard in Sri Lanka, which is a lower-middle-income country.

**Methods:**

A cross-sectional study with a 28-day post-discharge follow-up was carried out in 13 hospitals. Three hundred and forty-four patients who were diagnosed with STEMI or NSTEMI were recruited during the hospital stay. The pre-event QOL was measured using an interviewer-administered questionnaire which included the SF-36 QOL tool and medical details. Follow-up QOL was gathered using a questionnaire that was filled and posted back by participants. Of the recruited sample, 235 responded for the follow-up component. Analysis was conducted for associations between pre- and post-discharge QOL. Furthermore, comparisons were made between the STEMI and NSTEMI groups. Mann Whiney U test, Wilcoxon signed rank test and chi square test were used in the analysis.

**Results:**

The post-event QOL was lower in seven out of eight domains than the pre-event QOL (*p* < 0.05). The NSTEMI group had more risk factors and a significantly lower pre-event QOL for seven domains (*p* < 0.05), when compared to the STEMI group. For seven domains, the post-discharge QOL was not significantly different (*p* > 0.05) between the STEMI and NSTEMI groups. Post-discharge general-health QOL domain score was higher than the pre-MI score (*p* = 0.028) and was higher in the STEMI group compared to the NSTEMI group (*p* = 0.042). Regression analysis showed a significant beta coefficient between pre- and post-QOL for five domains in STEMI and for all domains in NSTEMI groups when adjusted for the disease severity. The R square values ranged from 12.3 to 62.3% for STEMI and 7.3 to 64.8% for NSTEMI.

**Conclusions:**

Pre-event QOL is lower in the NSTEMI group compared to the STEMI group. Patients do not regain the previous QOL within one month post-discharge. Post-discharge QOL can be predicted by the pre-event QOL for most domains.

## Background

The effect of chronic Non-Communicable Diseases (NCDs) on humans is multifaceted. There is a wide spectrum of such effects and they are more profound in the low- and middle-income (LMI) countries [1]. The chronic counterpart of the NCDs accounts for half of the Disability Adjusted Life Years (DALYs) in Sri Lanka [2]. Out of the chronic NCDs, the mortality and morbidity due to cardiovascular diseases are high in most parts of the world. Coronary heart disease has been a burden for developed countries as well as for developing countries [3–7]. Sri Lanka, being a LMI country, suffers from the consequences of these cardiovascular diseases [8]. Myocardial Infarction (MI) is one of the main acute critical effects which reflect the tip of the iceberg of chronic NCDs.

MI results from myocardial cell necrosis due to a sustained and significant ischemic stress. It results more frequently due to obstruction of blood flow due to plaques in coronary arteries or less frequently due to other mechanisms like coronary artery spasms. The exact definition of MI has been changed from time to time [9]. Following the 2008–09 revisions of World Health Organization’s definition and diagnostic criteria, MI was categorized into three categories called A, B and C. The “category A definition” is to be used in settings with no resource constraints [9]. ST-segment elevation myocardial infarction (STEMI) and non-ST segment elevation myocardial infarction (NSTEMI) can be differentiated from the electrocardiogram changes [10]. The incidence of STEMI and NSTEMI varies worldwide. As an example, in Kenya which is a LMI country, the STEMI: NSTEMI ratio was found to be 6:4 [11]. In contrast, a rising trend of NSTEMI is currently observed among the European countries [10]. In an observational study, the STEMI:NSTEMI ratio for a tertiary care medical unit in Sri Lanka was found to be 1:2 [8].

The severity of illness of MI can be described with the Thrombolysis in Myocardial Infarction (TIMI) risk score. TIMI risk score for NSTEMI is calculated by allocating a score out of seven [12]. The higher the TIMI risk score, the worse the severity of the disease. The TIMI risk score for STEMI is a score out of 14 [13]. Similar to NSTEMI, for STEMI too, the higher TIMI risk score provides a worse projection of mortality.

Quality of life (QOL) reflects the subjective perception of an individual regarding his or her position in the living contexts [14]. The impact of health condition on the QOL is referred to as health-related QOL [15]. Measuring QOL of cardiac patients is beneficial for several reasons [16]. One reason is that it reflects the success of the management in the patient’s point of view. Another is that it helps in identifying the physical or mental QOL-related problems of patients which are not assessed otherwise [17]. Examples include issues related to daily physical activities, limitations of social roles and the influence of pain on daily functions. When these QOL-related problems are identified, strategies can be included to address them in the ambulatory management plan of the MI survivor. Furthermore post-MI QOL can help in clinical decision making. Examples include deciding on the frequency of follow-up visits and the selection of patients to be followed up at the primary healthcare level institution.

There are many tools to measure QOL, such as generic measures (SF-36), utility measures (EQ-5D), etc. [18] Out of these, the conventional Short Form-36 (SF-36) covers a recall period of four weeks [19, 20]. Its questions cover eight scales, namely general-health, physical-functioning, role-limitation-physical, pain, vitality, social-functioning, role-limitation-emotional and mental health [19–21]. The SF-36 has been validated by several studies in Sri Lanka [22, 23]. It has been used in measuring the QOL among MI survivors globally [16, 24–27]. Furthermore, its validity as a good indicator has been assessed for measuring QOL in relation to different medical conditions [28–30].

Pre-admission factors have been identified as influencing the post-MI QOL [31, 32]. Retrospectively collected pre-admission QOL of the survivors of MI reflects such factors. Hence, pre-event QOL would reflect the real living circumstances of the patients prior to getting the MI. In general, the pre-admission QOL is found to be associated with post-discharge QOL of the patients who needed intensive treatment for medical conditions [33]. Literature on this association specifically in relation to MI is not commonly found.

There are not many follow-up studies that analyze the course of QOL following the occurrence of MI [34]. In the few global studies available, it has been shown that the QOL of the coronary patients is significantly impaired just after hospital discharge [35]. Some authors have shown a significant decrease of QOL among patients with myocardial infarction, even at three months following the event [24, 36]. Yet similar literature is scarce in relation to LMI countries and rarer in relation to Sri Lanka.

By comparing the pre-event-QOL between the STEMI and NSTEMI groups, the diagnostic prediction models can be developed with further evidence. If the pre-event QOL is found to be a predictor of post-event QOL, it would help in clinical decision making. When interventions are coupled to raise the community-based QOL, the health status of the patients following the occurrence of MI can be improved. By exploring the post-MI QOL, decisions can be taken on the frequency and prioritization of post-discharge management of patients, even during their hospital stay.

This study was conducted to describe the pre-event QOL and its influence on the post-event QOL among patients with STEMI and NSTEMI admitted to secondary and tertiary level government hospitals within the Western province of Sri Lanka.

## Methods

A cross-sectional study with a follow-up component was conducted in 13 government hospitals, in the Western province of Sri Lanka. Study population included the patients who were 16 years and above admitted with the diagnosis of STEMI or NSTEMI. The components mentioned under the Category A of the 2008–2009 revisions of the World Health Organization’s diagnostic criteria on MI were used as inclusion criteria. These included at least one value of the increased level of cardiac biomarkers (preferably troponin) with at least one of the following: symptoms of ischemia, ECG changes (new ST-T changes or new left bundle branch block), development of pathological Q waves and imaging evidence [9]. The demarcations of STEMI and NSTEMI were done based on European Society for Cardiology guidelines [10]. The participants with duration of stay less than 48 h and with any physical or mental condition (not related to the MI), perceived to be lowering the QOL, were excluded.

All patients who satisfied the eligibility criteria, who were admitted to the mentioned 13 government hospitals within the Western province, were invited for the study. The sample size was calculated using the formula n = z^2^ x sd^2^ / l^2^ using the highest standard deviation values in documented literature. With a design effect of 2, the needed sample size at the data analysis stage was 240 [37, 38]. With an assumed response rate of 70%, the minimum sample size needed at the data collection stage was calculated as 344. Therefore, this was the minimum sample size to be recruited within the hospital admission in order to describe the post-MI QOL. Data collection was initiated simultaneously in all the study settings on 1st of January 2015 and continued until the sample size was achieved in March 2015.

Data collection was carried out by the investigators and seven trained data collectors who were MBBS qualified graduates awaiting their medical internship. Data collectors visited the medical wards which were allocated to them out of the study settings daily; they verified the inclusion criteria, of the patients diagnosed with MI by the ward staff. The data collectors also interviewed the eligible patients in the first encounter—within day three or four of admission.

In the initial encounter with the participants, an interviewer-administered questionnaire and a data extraction form were used. The former included the socio-demographic details, past medical details of the participants and questions of the SF-36 QOL tool. The 36 questions of the QOL tool covered eight domains, which are physical activity (ten questions), role-limitation-physical (four questions), pain (two questions), general health (five questions), role-limitation-emotional (three questions), vitality (four questions), social functioning (two questions) and emotional wellbeing (five questions). The SF-36 focuses on the general health in relation to the 28 days prior to the date of reference. When answering this section, the participants were asked to focus on the 28 days prior to the current event that led to their hospital admission.

Having considered the potential risk prediction scores, the TIMI risk score was selected to be used in the present study, following the consultation of an expert panel which included experts in cardiology, general medicine and community medicine. The data extraction form included details on the eligibility criteria and for the calculations of the TIMI risk score. The TIMI risk scores for the NSTEMI group were categorized into six score levels. Those in the group who scored zero or one out of the total of seven marks were assigned to Level 1 and those who scored six or seven were assigned to Level 6. The scores 2,3,4 and 5 were assigned a level numerically equal to the score [12]. The TIMI risk scores for the STEMI group were categorized into 10 categories based on the criteria laid by Morrow and others in 2000 [13].

A self-administered questionnaire was given to the participants with a stamped envelope addressed to the principal investigator to collect data on the post-discharge day-28 QOL. The contact details were obtained and a reminder was given at the completion of 28 days to the participants.

The collected data were entered into a pre-designed sheet in Statistical package for Social Sciences (SPSS version 20). The characteristics of the study population were described using descriptive statistics. The eight domain scores were calculated using the recommended formulae [19]. Each domain was given a score out of 100. The eight domain scores were described using mean with standard deviation and median with interquartile range.

The normality assessment of the QOL scores was carried out with Box and Whisker diagrams, Q-Q plots and using the Kolmogorov–Smirnov test. These scores were found to be non-normally distributed. The missing items due to non-responders were in the form of unit-non-response and not as item-non-response. In analyzing for the possible non-response bias, the data available in the hospital-based component were used as auxiliary information. Characteristics of the responders versus non-responders based on the post-event data collection were compared [39–41]. A logistic regression model was created with the response status as the outcome variable to bring out any significant associations.

Past medical characteristics of the STEMI and the NSTEMI groups were compared using the chi square test. Scoring of the eight domains of SF-36 was carried out following the recommendations of its user manual and interpretation guide. Except for the item on “reported health transitions”, other 35 items were used in scoring of the domain scores, as shown in the Table 1 [19].

**Table 1:** Items used in the scoring of SF-36

Domain	Item numbers	Total number of items
General-health	1, 11a,11b, 11c,11d,	5
Physical- functioning	3a, 3b, 3c, 3d, 3e, 3f, 3g, 3h, 3i, 3j	10
Pain	7, 8	2
Role-limitation-physical	4a, 4b, 4c, 4d	4
Role-limitation-emotional	5a, 5b, 5c	3
Vitality	9a, 9e, 9g, 9i	4
Social-functioning	6, 10	2
Emotional- wellbeing	9b, 9c, 9d, 9f, 9h	5

Pre-event versus post-event QOL scores were compared using the non-parametric Wilcoxon Signed-Rank test. The QOL scores of STEMI and NSTEMI groups were compared using the non-parametric Mann-Whitney test [42]. Having completed the residual analysis, the linear regression technique was used to obtain the influence of the pre-event QOL on the post-event QOL. The associations were controlled for the severity of disease obtained from the TIMI risk score.

The quality of data was ensured by several means which included prior pre-testing of data collecting instruments, training of data collectors and cross verifications. Informed written consent was obtained. Data collections were undertaken without disturbing the patient management processes. Ethical clearance was obtained and administrative permissions were obtained prior to the initiation of data collection.

## Results

Out of the 344 participants, 74 (21.5%) were diagnosed with STEMI and 270 (78.5%) with NSTEMI. The response rates for the questions related to the eight domains ranged from 98 to 100%. Out of the recruited participants, 235 (68.3%) responded by sending back the post-MI questionnaire following one month of hospital discharge. The non-responders were either lost to follow-up or died following the MI. Analysis of the characteristics of the responders versus non-presponders revealed that there was no apparent non-response bias. In describing the pre-MI QOL, all responded participants were included. In the analysis of the associations between pre-MI QOL and the post-MI QOL, only the participants with both pre-MI QOL and post-discharge responses were included (i.e. pairwise deletion of missing records).

The median (IQR) of the age distribution of the total sample was 62.0 (54.0–70.0). When stratified, the STEMI group had the median (IQR) age of 59.5 (54.0–67.0) and the corresponding figures for the NSTEMI group were 63.0 (54.0–71.0). Of the participants who posted back the questionnaire, 163 were males and 72 females. In the STEMI group, the male to female ratio was 4:1, with 41 males and 11 females. In the NSTEMI group this ratio was 2:1, with 122 males and 61 females.

Some characteristics of the past medical history of the participants have been summarized in Table 2.

**Table 2:** Selected characteristics of the past medical history of participants

	STEMI		NSTEMI		Significance of the difference	Total sample	
	Yes N (%)	No N (%)	Yes N (%)	No N (%)		Yes N (%)	No N (%)
Diagnosed diabetes	30 (40.5)	44 (59.5)	119 (44.1)	151 (56.0)	χ^2^ = 0.587 *p* = 0.587	149 (43.2)	195 (56.5)
Diagnosed hypertensive	40 (54.1)	34 (45.9)	180 (66.7)	90 (33.3)	χ^2^ = 4.008 *p* = 0.045*	220 (64.1)	124 (35.9)
Diagnosed with High Cholesterol	21 (28.4)	53 (71.6)	126 (46.7)	144 (53.3)	χ^2^ = 9.804 *p* = 0.012*	147 (42.6)	197 (56.2)
Angina	28 (37.8)	46 (62.2)	146 (54.1)	124 (45.9)	χ^2^ = 6.125 *p* = 0.013*	174 (50.6)	170 (49.4)

*significant at 0.05 level, STEMI- ST elevation MI, NSTEMI-Non-ST elevation MI

Being diagnosed as a diabetic, hypertensive, with hypercholesterolemia and/or having angina were observed with a relatively higher frequency among the patients with NSTEMI. The differences in the prevalence between the latter three were statistically significant.

The pre-event and day-28 post-discharge QOL have been summarized and compared in Table 3. Except for the general-health domain, for all other seven domains of the SF-36, the post-discharge QOL scores were lower than the pre-event QOL. The highest decrease of QOL was observed in relation to the role-limitation-physical domain. All the seven differences were statistically significant (*p* < 0.001). The general-health domain showed the converse with a higher post-discharge figure (*p* < 0.05).

**Table 3:** Comparison of pre-event QOL scores and post-discharge QOL scores of the participants

	Pre-event QOL		Post-discharge QOL		Significance of the difference
	Mean (SD)	Median (IQR)	Mean (SD)	Median (IQR)	
General-health	47.55 (27.3)	45.00 (25.0–70.0)	50.99 (23.11)	45.00 (35.0–70.0)	*p* = 0.028*
Physical- functioning	64.45 (27.3)	70.00 (40.0–100.0)	54.33 (24.91)	50.00 (35.0–70.0)	*p* < 0.001**
Pain	69.5 (29.6)	77.5 (45.0–100.0)	65.72 (24.82)	67.5 (45.0–78.1)	*p* < 0.001**
Role-limitation-physical	51.6 (48.3)	75.00 (0.0–100.0)	22.13 (34.48)	0.00 (0.0–25.0)	*p* < 0.001**
Role-limitation-emotional	60.6 (47.1)	100.0 (0.0–100.0)	34.19 (41.83)	0.00 (0.0–66.7)	*p* < 0.001**
Vitality	57.6 (18.6)	60.00 (45.0–75.0)	54.91 (16.00)	55.00 (45.0–65.0)	*p* < 0.001**
Social-functioning	73.30 (25.3)	75.00 (50.0–100.0)	69.20 (21.44)	62.50 (62.5–87.5)	*p* < 0.001**
Emotional Wellbeing	60.50 (15.8)	60.00 (48.9–72.0)	56.67 (13.72)	56.00 (48.0–64.0)	*p* < 0.001**

*significant at 0.05 level, **significant at 0.001 level

Table 4 compares the pre-event QOL between the participants with STEMI and NSTEMI. For all the eight domains, the pre-event QOL values were higher in the STEMI group than the NSTEMI group. Except for the vitality domain, the differences were statistically significant for other seven domain scores. The highest difference between the two groups was observed in relation to the role-limitation-physical domain and the lowest difference was for the vitality domain.

**Table 4:** Comparison of pre-event QOL between participants with STEMI and NSTEMI

	STEMI		NSTEMI		Significance of the difference
	Mean (SD)	Median (IQR)	Mean (SD)	Median (IQR)	
General-health	55.84 (24.35)	55.00 (40.0–75.0)	45.37 (27.69)	45.00 (25.0–65.0)	*p* = 0.003*
Physical- functioning	73.21 (28.30)	85.00 (53.8–100.0)	62.10 (32.10)	65.00 (38.8–95.0)	*p* = 0.012*
Pain	78.62 (24.85)	88.75 (67.5–88.8)	66.89 (30.34)	67.5 (45.0–100.5)	*p* = 0.004*
Role-limitation-physical	68.49 (44.10)	100.00 (0.0–100.0)	46.83 (48.37)	25.0 (0.0–100.0)	*p* = 0.002*
Role-limitation-emotional	73.97 (42.03)	100.00 (33.3–100.0)	56.78 (47.89)	100.0 (0.0–100.0)	*p* = 0.006*
Vitality	60.89 (16.94)	60.00 (48.3–75.0)	56.75 (19.00)	55.00 (45.0–70.0)	*p* = 0.113
Social-functioning	79.28 (21.97)	75.00 (75.0–100.0)	71.65 (26.01)	75.00 (50.0–100.0)	*p* = 0.028*
Emotional Wellbeing	65.30 (14.15)	64.00 (56.0–76.0)	59.19 (15.96)	60.00 (48.0–72.0)	*p* = 0.007*

*Significant at 0.05 level, STEMI- ST elevation MI, NSTEMI-Non-ST elevation MI

The comparison between post-discharge QOL of the STEMI and NSTEMI groups is summarized in Table 5. Even though the QOL domain scores were relatively lower for the NSTEMI group, a statistically significant difference was observed only for the general-health domain. The highest difference was observed for the role-limitation-physical domain and the lowest for the vitality domain.

**Table 5:** Comparison of post-discharge QOL between participants with STEMI and NSTEMI

	STEMI		NSTEMI		Significance of the difference
	Mean (SD)	Median (IQR)	Mean (SD)	Median (IQR)	
General-health	55.99 (19.27)	50.00 (45.0–70.0)	49.78 (23.80)	45.00 (35.0–70.0)	*p* = 0.042*
Physical- functioning	59.14 (21.50)	57.50 (40.0–78.8)	53.24 (25.46)	50.00 (35.0–70.0)	*p* = 0.088
Pain	68.89 (22.65)	67.5 (45.0–87.5)	65.04 (25.26)	67.5 (45.0–77.5)	*p* = 0.403
Role-limitation-physical	31.25 (43.41)	0.00 (0.0–93.8)	19.64 (35.39)	0.00 (0.0–25.0)	*p* = 0.079
Role-limitation-emotional	39.10 (41.60)	33.33 (0.0–100.0)	32.96 (41.94)	0.00 (0.0–66.7)	*p* = 0.199
Vitality	57.26 (13.83)	55.00 (47.5–65.8)	54.44 (16.33)	55.00 (45.0–65.0)	*p* = 0.258
Social-functioning	72.41 (18.07)	75.00 (62.5–87.5)	68.58 (21.95)	62.50 (62.5–87.5)	*p* = 0.172
Emotional Wellbeing	59.32 (12.27)	60.00 (52.0–68.0)	55.95 (14.06)	56.00 (48.0–64.0)	*p* = 0.127

*Significant at 0.05 level, STEMI- ST elevation MI, NSTEMI-Non-ST elevation MI

Tables 6 and 7 show the stratified analysis of the pre- versus post-discharge QOL and the influence of the pre-event QOL on the post-discharge QOL for STEMI and NSTEMI groups using the Wilcoxon signed rank test. Except for the general-health domain, for all other domains, there is a reduction of the QOL scores following the event. The higher reductions have been observed for the two role-limitation domains and the social-functioning domain. For the seven domains which show a reduction of QOL scores, the differences have been statistically significant (Table 6).

**Table 6:** Comparison of pre-event QOL versus post-event QOL and the influence of pre-event QOL on post-discharge QOL adjusted to severity among patients with STEMI

	Pre-QOL	Post QOL	Significance of Difference	Beta coefficient	Significance	R Square
General-health	55.84	55.99	*p* = 0.653	0.625	*p* < 0.001**	0.623
Physical- functioning	73.21	59.14	*p* < 0.001**	0.335	*p* = 0.002*	0.212
Pain	78.61	68.89	*p* = 0.004*	0.169	*p* = 0.175	0.071
Role-limitation-physical	68.49	31.25	*p* < 0.001**	0.050	*p* = 0.733	0.005
Role-limitation-emotional	73.97	39.10	*p* = 0.001**	0.042	*p* = 0.773	0.004
Vitality	60.89	57.26	*p* = 0.012*	0.465	*p* < 0.001**	0.339
Social-functioning	79.28	21.97	*p* = 0.002*	0.297	*p* = 0.020*	0.123
Emotional Wellbeing	65.30	59.32	*p* < 0.001**	0.566	*p* < 0.001**	0.388

*significant at 0.05 level, **significant at 0.001 level

**Table 7:** Comparison of pre-event QOL versus post-event QOL and the influence of pre-event QOL on post-event QOL adjusted to severity among patients with NSTEMI

	Pre-QOL	Post QOL	Significance of difference	Beta coefficient	Significance	R Square
General-health	45.37	49.78	*p* = 0.004*	0.673	*p* < 0.001**	0.648
Physical- functioning	62.10	52.24	*p* < 0.001**	0.494	*p* < 0.001**	0.296
Pain	66.89	65.04	*p* = 0.008*	0.358	*p* < 0.001**	0.164
Role-limitation-physical	46.83	19.64	*p* < 0.001**	0.203	*p* < 0.001**	0.073
Role-limitation-emotional	56.78	32.96	*p* < 0.001**	0.333	*p* < 0.001**	0.142
Vitality	56.75	54.44	*p* = 0.004*	0.490	*p* < 0.001**	0.326
Social-functioning	71.65	68.58	*p* < 0.001**	0.445	*p* < 0.001**	0.257
Emotional Wellbeing	59.19	55.95	*p* < 0.001**	0.536	*p* < 0.001**	0.395

*significant at 0.05 level, **significant at 0.001 level

Except for the general-health domain, which has shown an increased value, reductions have been observed in the other seven QOL domains in the NSTEMI group as far as the pre-event and post-discharge scores are concerned (Table 7). The differences between the pre- and post-event QOL scores were statistically significant for the eight domains.

The mean (SD) of the TIMI risk scores for STEMI group was 3.88 (1.34) and for the NSTEMI group this value was 2.95 (1.34). When adjusted for the severity of the disease (TIMI risk score), the beta coefficient of the regression model (getting post-discharge QOL as the dependent variable and pre-event QOL as the independent variable) was observed to be statistically significant for five domains in the STEMI group. For all the domains, the pre-event QOL has given a prediction for the post-discharge QOL in the positive direction. General-health (0.625), emotional-wellbeing (0.566) and vitality (0.465) have shown the three highest values for the coefficient. Out of the models with significant beta coefficients, the explanation of variability of the post-discharge QOL (R square) has varied between 12.3% (social-functioning) and 62.3% (general-health).

When the regression analysis was performed for the NSTEMI group, all the beta coefficients of the models predicting the post-discharge QOL based on the pre-event QOL (when adjusted for the TIMI risk score) were observed to be statistically significant (*p* < 0.001). The R square ranged from the lowest value of 7.3% (for role-limitation-physical) to the highest value of 64.8% (for general-health).

## Discussion

This study shows that the post-event 28-day QOL is lower than the pre-event QOL (*p* < 0.05) in patients with MI, except in the general-health domain. Patients who later developed NSTEMI were susceptible to more risk factors compared to patients who developed STEMI. Pre-event QOL of patients with NSTEMI was significantly lower in seven domains compared to that for patients with STEMI. The post-discharge QOL for seven domains between STEMI and NSTEMI groups were not significantly different. The post-discharge score of the exceptional general-health domain was higher in the STEMI group. Regression analysis showed a significant beta coefficient between pre- and post-QOL for five domains in STEMI and for all domains in NSTEMI groups when adjusted for disease severity. The R square values ranged from 12.3 to 62.3% for STEMI and 7.3 to 64.8% for NSTEMI.

Sri Lanka, although classified as a LMI country, has a free healthcare system and has notably improved health parameters compared to many other similar settings. The ongoing demographic and epidemiological transitions have made the inhabitants more vulnerable to NCDs [2, 43]. Sri Lanka is in the process of taking invaluable steps to combat the potential threat of a chronic NCD epidemic [44]. Patients with chronic NCDs are at risk of experiencing an acute critical event due to the underlying life-long disease processes [45].

In order to provide comprehensive healthcare for control of chronic NCDs, the health system should be vigilant on the occurrence, management and prevention of these events. The evaluation of the pre-event QOL would give a picture of the day-today living at the home-based level as well as the health inequalities that persist in the community. The QOL at one month following the discharge from hospital would reveal such information plus the quality of management of a condition [25].

The Western province comprises approximately 28.8% of the total population of Sri Lanka [46]. Following MI, there is a higher chance for the patients to be admitted to one of the study settings which are secondary and tertiary care hospitals in the Western province. Hence, the study settings reflect more or less the actual dynamics in relation to the management of MI patients in the whole Western province.

By utilizing SF-36, the pre-event QOL was assessed. This method of measuring pre-admission QOL retrospectively is similar to the methodology of Failde & Soto [36] and Hofhuis et al. [47]. Even though the pre-event QOL was initially planned to be collected through a self-administered questionnaire, in the pre-testing, patients preferred it to be interviewer-administered.

It can be assumed that at the time of the hospital discharge, patients would have had health conditions suitable to dwell in the community setting. The follow-up QOL was measured at approximately one month following hospital-exit point. Because SF-36 is also applicable for a period of 28 days, it becomes an ideal tool for this purpose [20]. Furthermore, the findings of generic QOL tools can be used to compare the QOL between different groups [48].

The participants with STEMI were relatively younger than those with NSTEMI [49–51]. Age has been identified as a determinant of QOL in Sri Lanka and in global literature [52, 53]. The comparative results were seen in relation to the critically ill patients in the global literature too [54]. This may be due to the higher prevalence of other NCDs in the NSTEMI group (Table 2) [49–51, 55]. The higher prevalence of the NCDs and the other higher-age-associated factors would have contributed to this reduction of pre-event QOL in the NSTEMI group [56, 57] (Table 4). Furthermore, these facts reflect the relatively chronic onset of NSTEMI compared to STEMI which is highlighted in global literature as well [58].

The negative impact of the lower pre-event scores of NSTEMI on the community is worsened due to its higher relative incidence. It was observed that the STEMI: NSTEMI ratio was approximately 1: 5. This is an exaggeration of the documented local prevalence mentioned by Medagama and others in 2015 [8]. It is, however, on par with the current trend of European countries and in contrast to the prevalence recorded in some African countries [10, 11].

There has been no notable distinction between the domains belonging to the physical health component and those that belong to the mental health component. This is in contrast to the results of Pappa et al. [59] which showed contradictory findings.

The post-discharge 28-Day QOL showed a significant reduction compared to pre-event QOL (Table 3), except for the general health domain. This is in agreement with the documented global literature [32]. Once stratified by the MI category (Tables 6 and 7), similar results were observed for seven domains out of eight. Yet for most of the domains (Table 5), the post-discharge QOL did not show a significant difference between the STEMI and NSTEMI groups. These phenomena give rise to two important hypotheses. One is that even after almost one month following hospital discharge, the patients with MI apparently had not re-gained their previous health status [58]. Second inference is that the measures of improving the QOL should be applied irrespective of the MI category [53, 58].

The general-health domain score, unlike in other domains, was statistically significantly higher among the NSTEMI group compared to that in the STEMI group (Table 5). In literature, it is documented that the NSTEMI group has less “health reserve” due to the higher age and prevalence of comorbid factors. It has been mentioned as an explanation of the NSTEMI group having a higher post-event QOL than the STEMI group [58]. Furthermore, the NSTEMI group had a higher QOL score for general-health in the post-event assessment than its pre-event score. This can be explained with the five variables assessed under the particular domain in SF-36 [20]. They cover the aspects of general perception of health, perception of getting sick more easily than others, perception of being as healthy as others and perception of worsening health. The prevalent comorbidities mentioned in Table 1 would have been attended to during the hospital stay. This would have contributed towards improving the post-event general-health domain of the NSTEMI group compared to the pre-event QOL.

For five domains in the STEMI category and for all the domains of the NSTEMI category, the regression models revealed a positive beta coefficient in predicting the post-discharge QOL by the respective pre-event QOL (Tables 6 and 7). This has been analyzed separately, as the TIMI risk scores for the two groups are calculated differently. This fact points towards the possibility of predicting the post-discharge QOL using the pre-event QOL during hospital stay. With further research, prediction models could be established with the inclusion of more parameters. These models would be useful especially in resource-poor settings in deciding on the follow-up care of the patients.

There were several limitations to this study. One was that the data were obtained after 48 h of hospital admission, for ethical reasons. Hence, the patients who died during this period were not included in the study. Expert guidance was used in excluding patients with conditions with concomitant conditions which could potentially affect the QOL. But a potential element of selection bias could not be totally excluded by this. Furthermore, mortality was not analyzed. These were accounted for when interpreting the findings. Another limitation was that in describing the QOL, the eight domains were described without adhering to the next optional step of calculation of the two summary scores (i.e., physical summary score and the mental summary score). This was because the context specific population coefficients were not available. Hence, the eight domain scores were calculated in order to preserve the internal validity. The pre-event QOL was measured by an interviewer-administered questionnaire while the post-discharge measurement was self-administered. The STEMI and NSTEMI groups were not identical in relation to the number of participants, their age distribution, and gender distribution. In order to minimize any bias due to this, the findings of the two groups have been presented separately with stratification.

Another limitation was that, even though the basic facilities needed for the management of MI are available in all 13 settings, interventions like Percutaneous Coronary Interventions were not available in many of the settings. When the admitted patients needed these interventions, they were transferred to the settings which had those services. At the hospital discharge, all were able to live a community-based life. The post-MI QOL was calculated 28 days following the hospital discharge, thereby minimizing the influence of the intervention on the post-MI QOL. Immediate baseline QOL measurements were not taken. Pair-wise deletion was done as the apparent pattern of missingness was “ignorable”. But since the potential of bias cannot be totally excluded, this must be concerned in interpreting the findings. It must be further stated that since the findings are based on a provincial community cohort in a lower income country, the results may not be applicable to other broader settings.

## Conclusions

When compared to the pre-event QOL, there is a significant reduction of QOL at day 28 after hospital discharge in relation to seven domains covered by the SF-36 among patients who experienced a MI. Almost similar findings are observed when stratified according to the MI category. Pre-event QOL is relatively higher among the STEMI group compared to NSTEMI group. For seven domains, the post-discharge QOL was not significantly different between these groups. The post-discharge general-health QOL score was significantly higher in the STEMI group. Pre-event QOL can predict the post-discharge QOL with significant beta coefficient values in five domains for the STEMI group and for all domains in the NSTEMI group. Attempts must be made to improve the community-based QOL in order to have favorable post-MI QOL. It would be beneficial to encourage more research on the QOL of MI survivors, the findings of which facilitate the clinical decision making especially in resource poor settings.
